# Myokines as potential mediators of changes in glucose homeostasis and muscle mass after bariatric surgery

**DOI:** 10.3389/fendo.2025.1554617

**Published:** 2025-03-18

**Authors:** Laura Orioli, Jean-Paul Thissen

**Affiliations:** ^1^ Research Laboratory of Endocrinology, Diabetes, and Nutrition, Institute of Experimental and Clinical Research, UCLouvain, Brussels, Belgium; ^2^ Department of Endocrinology and Nutrition, Cliniques Universitaires Saint-Luc, Brussels, Belgium

**Keywords:** glucose homeostasis, myokines, myostatin, muscle mass, obesity, sarcopenic obesity, sarcopenia, type 2 diabetes

## Abstract

Myokines are bioactive peptides released by skeletal muscle. Myokines exert auto-, para-, or endocrine effects, enabling them to regulate many aspects of metabolism in various tissues. However, the contribution of myokines to the dramatic changes in glucose homeostasis and muscle mass induced by bariatric surgery has not been established. Our review highlights that myokines such as brain-derived neurotrophic factor (BDNF), meteorin-like protein (Metrnl), secreted protein acidic and rich in cysteine (SPARC), apelin (APLN) and myostatin (MSTN) may mediate changes in glucose homeostasis and muscle mass after bariatric surgery. Our review also identifies myonectin as an interesting candidate for future studies, as this myokine may regulate lipid metabolism and muscle mass after bariatric surgery. These myokines may provide novel therapeutic targets and biomarkers for obesity, type 2 diabetes and sarcopenia.

## 1 Introduction

Skeletal muscle was first proposed as an endocrine organ more than two decades ago, based on evidence that human skeletal muscle releases interleukin (IL)-6 into the circulation during exercise (1, 2). Since then, numerous bioactive molecules, including proteins, exosomes, metabolites and microRNAs, have been identified as part of the human muscle secretome using computational and -omics approaches (2–6). Specifically, proteins secreted by skeletal muscle are called myokines (7, 8). More than 600 myokines have been identified in conditioned media from human muscle cells using mass spectrometry, the most accurate and specific method currently available for secretome analysis (9). Myokines exert auto-, para- or endocrine effects, enabling them to regulate many aspects of metabolism in various tissues (2, 8, 10, 11). Some myokines are released in response to muscle contraction, leading to the concept of exerkines (12–15). Emerging evidence suggests that certain myokines/exerkines may contribute to the metabolic and cardiovascular benefits of exercise by facilitating communication between muscle and other organs, such as the liver, adipose tissue, heart, brain, and pancreas (2, 15, 16). However, the exact mechanisms underlying the actions of myokines and the extent of their influence remain areas of ongoing research.

Obesity and type 2 diabetes (T2D) are closely related conditions that represent significant global health problems due to their increasing prevalence and comorbidities (17). Both conditions are linked to alterations in muscle metabolism and function, which may contribute to age-related decline in muscle health (18–20). The underlying mechanisms are complex and include structural and metabolic changes in muscle tissue, altered myogenesis, fat infiltration (i.e. myosteatosis) and muscle inflammation (20–26). Together, these disorders can lead to a vicious cycle of muscle fatigue, decreased physical activity and energy expenditure, further fat gain, ultimately leading to sarcopenic obesity (18–20). The contribution of myokines to altered muscle metabolism and function in people with obesity and T2D is not well understood.

Bariatric surgery is effective for treating obesity and T2D, leading to the concept of metabolic surgery (27–29). Bariatric surgery has many beneficial effects on skeletal muscle metabolism and function. Most importantly, bariatric surgery improves or even reverses muscle insulin resistance after significant weight loss (30–32). However, bariatric surgery induces a significant loss of muscle mass, mainly due to profound calorie and protein restriction (33, 34). Despite reduced muscle mass, muscle function and quality (i.e. muscle strength to muscle mass ratio) improve after bariatric surgery (35, 36). The underlying mechanisms include extensive changes in the insulin signaling cascade, improved muscle oxidative capacity and mitochondrial function, as well as intramyocellular lipid depletion, reduced muscle inflammation and fibrosis (32, 37–39). The contribution of myokines to changes in glucose homeostasis, muscle mass and function after bariatric surgery remains poorly understood.

In this review, we will comprehensively discuss the potential role of myokines in changes in glucose homeostasis and muscle mass after bariatric surgery.

## 2 An overview of changes in myokines in obesity and type 2 diabetes

The muscle secretome is altered in people with obesity and T2D. These alterations include an increased release of ILs, including IL-6, IL-8 and IL-15, and pro-inflammatory cytokines, such as tumor necrosis factor alpha (TNF-a) and monocyte chemotactic protein-1 (MCP-1) (40, 41). Increased release of follistatin (FST) and myostatin (MSTN), which have opposing effects on muscle mass, has also been reported (40, 41). Altered myokine secretion may be an intrinsic property of muscle in people with obesity and T2D due to genetic or epigenetic factors (42). Alternatively, altered myokine secretion may result from muscle exposure to obesity-related metabolic factors, such as lipotoxicity and inflammation, which impair muscle insulin sensitivity (43–45). In addition, insulin itself regulates myokine secretion by muscle cells directly (46) and indirectly by increasing the expression of type IIx muscle fibers, whereas some myokines are type II fiber-specific (e.g., IL-6, angiogenin, osteoprotegerin) (47–50). Therefore, hyperinsulinemic states such as obesity and T2D are likely to affect myokine expression and secretion.

In turn, altered myokine secretion in obesity and T2D is likely to affect glucose homeostasis and muscle mass. Indeed, conditioned medium from insulin-resistant human myotubes blunts glucose-stimulated insulin secretion (GSIS) and increases beta cell apoptosis (43). Similarly, conditioned medium from inflamed myotubes induces muscle cell inflammation and insulin resistance (51). Pro-inflammatory myokines such as IL-1β, IL-6, IL-8, TNF-a, C-C motif chemokine 5 (CCL5), MCP-1, and C-X-C motif chemokine 10 (CXCL10) are likely mediators of these detrimental effects on beta cells and muscle cells (43, 51). In addition, conditioned medium from myotubes derived from humans with extreme obesity inhibits proliferation of C2C12 myoblasts, an effect that is abolished in the presence of an anti-MSTN antibody (41). On the other hand, increased expression and secretion of myokines such as fractalkine, osteoprotegerin, chitinase-3-like protein 1, irisin, and FST by insulin-resistant inflamed human myotubes may represent an autoprotective mechanism counteracting inflammation, insulin resistance, beta cell dysfunction, and sarcopenia (8, 40, 41, 43, 52). Thus, some myokines may contribute to the changes in glucose homeostasis and muscle mass observed in people with obesity and T2D.

## 3 Changes in myokines following bariatric surgery related to glucose homeostasis and muscle mass

The four most common types of bariatric surgery were included in this review: laparoscopic adjustable gastric banding, sleeve gastrectomy, Roux-en-Y gastric bypass, and biliopancreatic diversion. These procedures will be referred to hereafter as bariatric surgery.

**Figure 1:**
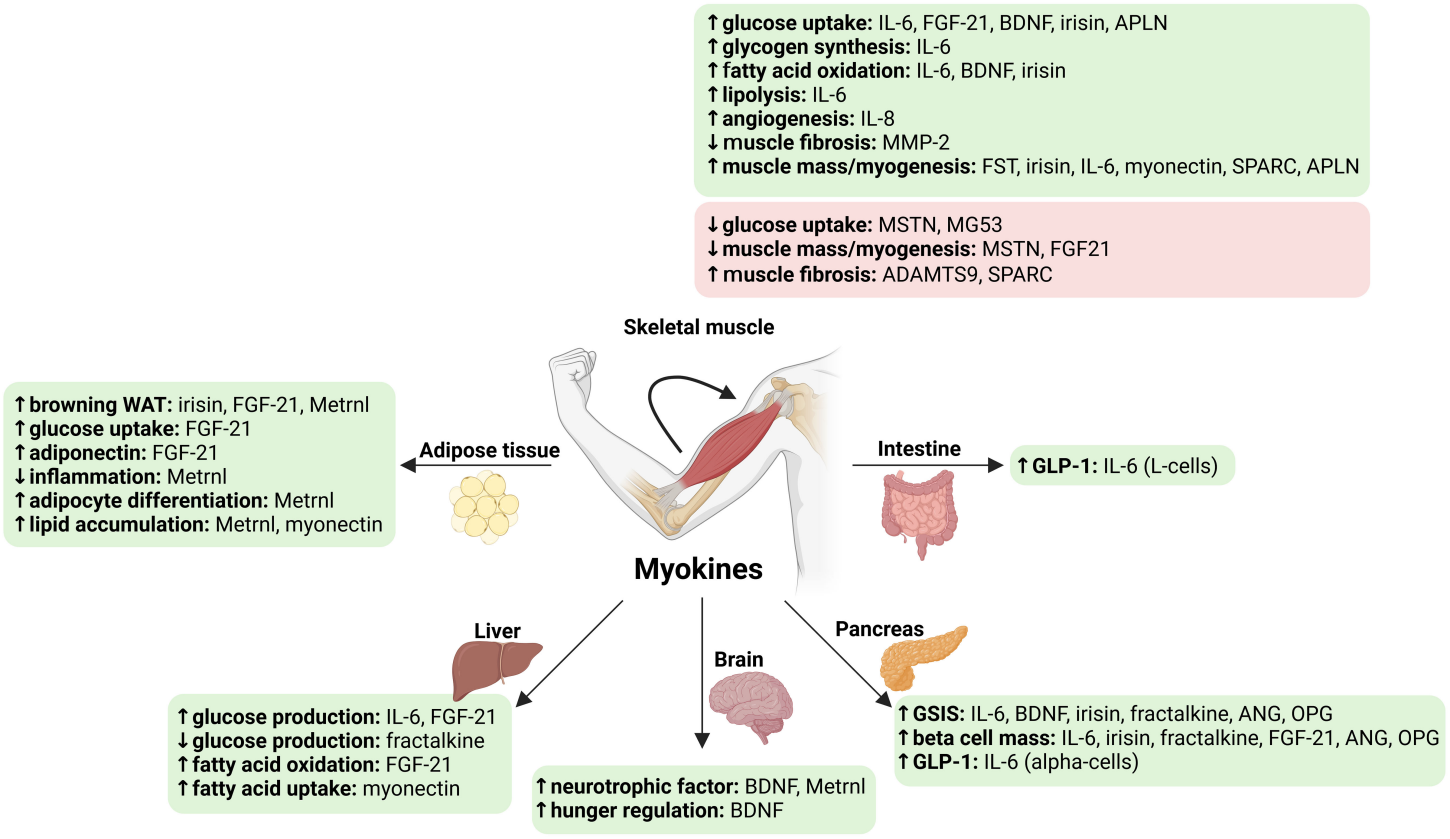
Main known effects of myokines altered by bariatric surgery on glucose homeostasis and muscle mass. ↑, increase; ↓, decrease. Green, beneficial effects; Red, detrimental effects. ADAMTS9, ADAM metallopeptidase with thrombospondin type 1 motif 9; ANG, angiogenin; APLN, apelin; BDNF, brain-derived neurotrophic factor; GSIS, glucose-stimulated insulin secretion; FGF21, fibroblast growth factor 21; FST, follistatin; GLP-1, glucagon-like peptide 1; IL, interleukin; Metrnl, meteorin-like protein; MG53, mitsugumin (or Tripartite motif containing 72, TRIM72); MMP-2, 72 kDa type IV collagenase; MSTN, myostatin; OPG, osteoprotegerin; SPARC, secreted protein acidic and cysteine-rich (osteonectin); WAT, white adipose tissue. Created in https://BioRender.com.

Overall, bariatric surgery alters the circulating levels and muscle expression of specific myokines (53–56). The main effects of these myokines on metabolism and muscle mass are illustrated in **Figure 1**. Changes in some of these myokines may be associated with improved glucose homeostasis after bariatric surgery, which includes improved systemic and tissue-specific insulin sensitivity and beta-cell function (**Table 1**) (30, 57, 58). In addition, some of these myokines may be associated with changes in muscle mass after bariatric surgery (**Table 1**).

**Table 1:** Changes in myokines after bariatric surgery and their potential contribution or association to changes in glucose homeostasis and muscle mass after bariatric surgery based on reported associations or known effects on glucose homeostasis or muscle mass.

Myokine	Changes after bariatric surgery	Potential effects of changes in myokines
Fractalkine	↑ circulating levels	↑ GSIS
	↑ muscle mRNA	↑ muscle insulin sensitivity through shift in substrate use toward glucose in muscle
BDNF	↓ circulating levels	↓ anorectic stimulus association with ↓ compensatory hyperinsulinemia
	↑ muscle mRNA	↑ muscle insulin sensitivity ↑ muscle strength/myogenesis
MSTN	↓ circulating levels	↑ muscle insulin sensitivity ↑ muscle mass
	↓ muscle mRNA	
Irisin	↓; ↔; ↑ circulating levels	? changes in energy expenditure
	↓ muscle mRNA	↓ muscle mass
IL-6	↓; ↔ circulating levels	↑ muscle insulin sensitivity ↑ muscle strength/myogenesis
	↑ muscle mRNA	
FGF21	↑ circulating levels	↑ muscle insulin sensitivity ↓ muscle mass
Metrnl	↓; ↔; ↑ circulating levels	? changes in energy expenditure ↑ Metrnl inversely associated with ↓ HOMA-IR
Myonectin	↔; ↑ circulating levels	↑ muscle insulin sensitivity ↑ muscle mass
APLN	↓ circulating levels	? muscle insulin sensitivity ↓ muscle mass
	↔ muscle mRNA	
SPARC	↓ circulating levels	↑ muscle insulin sensitivity ↓ muscle mass association with ↓ HOMA-IR
MMP-2	↓; ↑ circulating levels	? muscle insulin sensitivity through ECM remodeling
FST	↓; ↔; ↑ circulating levels	Surgery alone with exercise: counter-regulation of MSTN
	Surgery alone: ↓ muscle mRNA Surgery with exercise: ↑ muscle mRNA	
ADAMTS9	↑ muscle mRNA	? muscle insulin sensitivity through ECM remodeling
MG53	↑ muscle mRNA	? muscle insulin sensitivity
ANG	↑ muscle mRNA	↑ GSIS
OPG	↑ muscle mRNA	↑ GSIS
ANG	↑ muscle mRNA	↑ GSIS
IL-8	↔ circulating levels	? muscle insulin sensitivity though muscle angiogenesis
	(↑ secretion by myotubes)	

↑, increase/increased; ↓, decrease/decreased; ADAMTS9, ADAM metallopeptidase with thrombospondin type 1 motif 9; ANG, angiogenin; APLN, apelin; BDNF, brain-derived neurotrophic factor; GSIS, glucose-stimulated insulin secretion; FGF21, fibroblast growth factor 21; FST, follistatin; IL, interleukin; Metrnl, meteorin-like protein; MG53, mitsugumin (or Tripartite motif containing 72, TRIM72); MMP-2, 72 kDa type IV collagenase; MSTN, myostatin; OPG, osteoprotegerin; SPARC, secreted protein acidic and cysteine-rich (osteonectin).

### 3.1 Fractalkine

Fractalkine, also known as C-X3-C motif chemokine ligand 1 (CX3CL1), is the only member of the CX3C chemokine family (59). Fractalkine is initially synthesized in a plasma membrane-bound form, and its soluble form is released by enzymatic cleavage (59). Fractalkine is the specific ligand for a G protein-coupled receptor called CX3CR1, which mediates its effects on chemotaxis, cell adhesion, and increased cell survival during inflammation (59). Fractalkine is secreted by human muscle cells, although it has a low tissue specificity (12, 43, 60, 61). Contraction upregulates fractalkine expression in human muscle cells and tissue and increases its circulating levels (12–14).

Fractalkine may have beneficial metabolic effects, particularly on beta cells. Indeed, CX3CR1 knockout mice have impaired GSIS, which is also observed in isolated pancreatic islets from these mice (62). In contrast to CX3CR1 knockout mice, wild-type mice treated with fractalkine show improved glucose tolerance and GSIS, while fractalkine also potentiates GSIS in mouse and human islets (62). Similarly, chronic administration of a long-acting form of fractalkine improves glucose tolerance in obese rodents, as evidenced by improved GSIS and hepatic insulin sensitivity (63). In addition, fractalkine protects rodent beta cells from apoptosis and GSIS from pro-inflammatory cytokines (59, 62). Fractalkine expression in islets is reduced in mice fed a high-fat diet and in obese mice, suggesting that reduced fractalkine signaling may contribute to beta cell dysfunction in people with obesity and T2D (62). In addition, administration of purified fractalkine directly into skeletal muscle of mice modulates mitochondrial metabolism and shifts substrate preference toward glucose, again suggesting that fractalkine signaling is involved in the regulation of muscle insulin sensitivity (64). These promising data in rodent models have led to the identification of several compounds such as ZINC000032506419 with a strong binding affinity for CX3CR1 (65). However, the effects of these compounds on glucose homeostasis are currently unknown. Data on fractalkine in humans are variable. Some studies have reported higher circulating fractalkine levels in people with obesity and T2D, while others have not (8, 66, 67). Data on fractalkine expression in muscle of people with obesity and T2D are also conflicting (8).

Nevertheless, we showed that circulating fractalkine levels increased by 7% three months after bariatric surgery, while fractalkine mRNA expression increased by 73% in the vastus lateralis, suggesting that muscle may contribute to the changes in circulating levels (54). Furthermore, the amplitude of these changes is comparable to those observed at circulatory and muscle levels after acute exercise, suggesting a potential beneficial effect of increased fractalkine on glucose homeostasis after bariatric surgery similar to exercise (13–15). To our knowledge, our study is among the first to explore the relationship between bariatric surgery and fractalkine. However, we did not find an association between changes in circulating or muscle fractalkine and changes in glucose homeostasis as assessed by the Homeostasis Model Assessment (HOMA) test (54). We believe future studies should evaluate the relationship between fractalkine and glucose homeostasis using more sophisticated methods to assess changes in muscle insulin sensitivity and beta cell function after bariatric surgery (30, 58).

### 3.2 Brain-derived neurotrophic factor

Brain-derived neurotrophic factor (BDNF) is mainly expressed in the central and peripheral nervous system, where it acts as a potent neuronal survival factor and modulator of synaptic plasticity (60, 61, 68). In humans, 70-80% of circulating BDNF originates from the brain (8). However, BDNF is also secreted by human muscle cells, which qualifies BDNF as a myokine (5, 12, 68). Contraction upregulates BDNF mRNA expression in human muscle tissue and cells (12, 14, 68–71). In humans, acute exercise increases circulating BDNF levels, whereas chronic exercise training does not change, increases, or even decreases circulating BDNF levels (15, 71). There are two major isoforms of the mammalian BDNF receptor TrkB, including TrkB.T1, which is expressed in the human pancreas (68).

**Figure 2:**
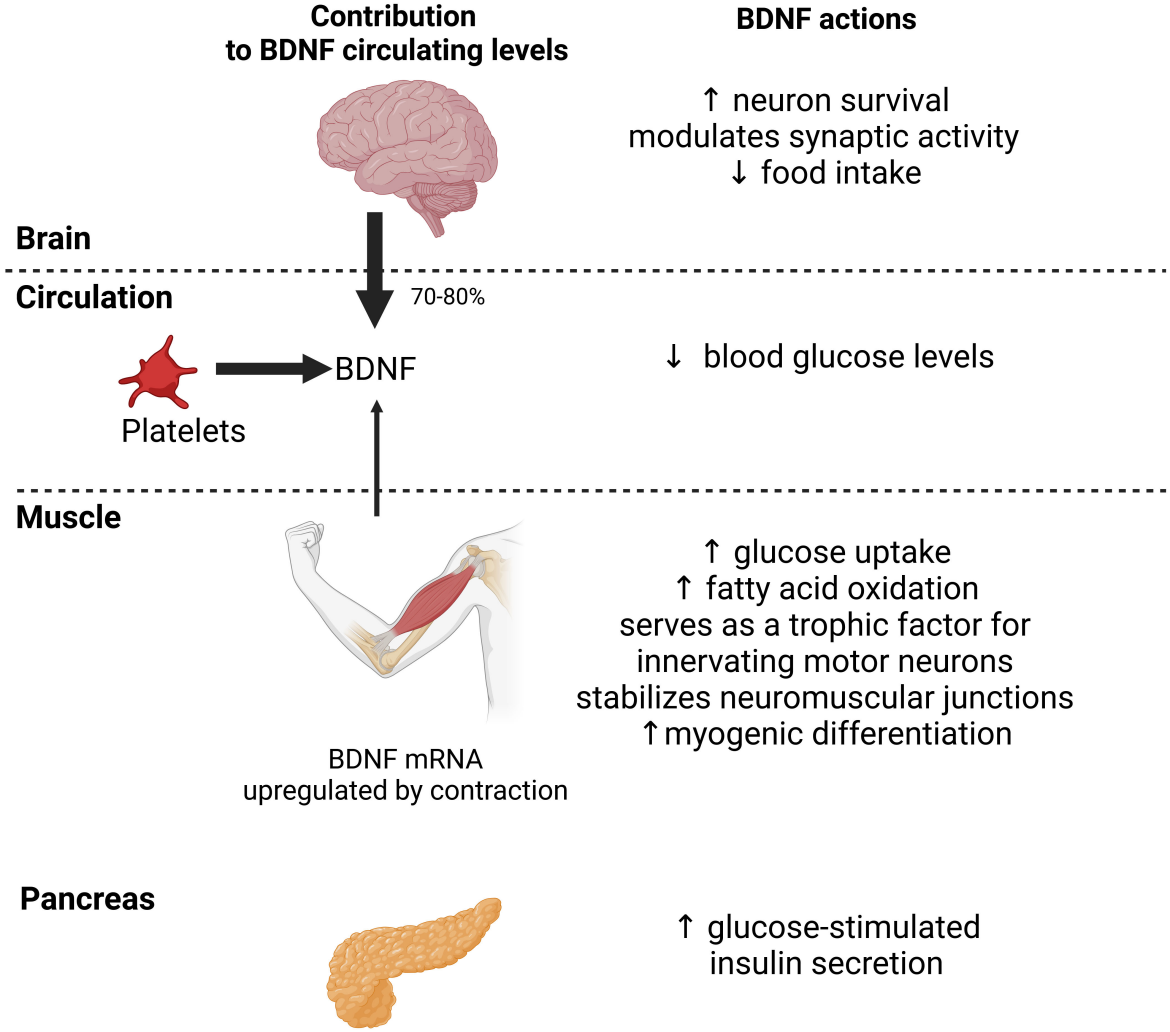
Actions of central and myocyte-specific brain-derived neurotrophic factor (BDNF) on glucose homeostasis. In humans, most circulating BNDF originates from the brain (70-80%) and platelets. Muscle also contributes to circulating levels. BDNF expression is upregulated in muscle by acute exercise which increases BDNF circulating levels. BDNF actions at the central and peripheral levels may decrease food intake, stimulate glucose uptake and fatty acid oxidation by muscle, and enhance glucose-stimulated insulin secretion by pancreas. These actions should collectively improve glucose homeostasis. Black arrows indicate the relative contribution of the brain, platelets and muscle to BDNF circulating levels.

BDNF has beneficial metabolic effects on skeletal muscle and beta cells (**Figure 2**). Systemic BDNF injections in mouse models of obesity and diabetes decrease blood glucose levels by enhancing muscle glucose uptake (68, 72, 73). However, BDNF has no direct effect on glucose uptake in muscle cells *in vitro* (8, 74). In contrast, BDNF increases fatty acid oxidation in muscle by activating AMPK (8, 69). In addition, pancreatic BDNF-TrkB.T1 signaling increases GSIS in human pancreatic islets (68). Muscle-specific BDNF knockout mice have reduced circulating BDNF levels, demonstrating that muscle is also a source of circulating BDNF (68). In addition, these mice exhibit glucose intolerance and blunted GSIS, suggesting that BDNF is a myokine that acts in an endocrine manner on beta cells (68). Finally, mice and humans heterozygous for mutations that inactivate BDNF or TrkB develop hyperphagic obesity (68), suggesting a role for BDNF in inhibiting hunger (**Figure 2**).

BDNF has long been thought to serve only as a retrograde trophic factor for innervating motor neurons and stabilization of neuromuscular junctions in muscle (71, 75). However, mouse and cell models have shown that BDNF is expressed in satellite cells promoting myogenic differentiation (75) (**Figure 2**). Hence, muscle-damaging exercise in healthy people is associated with increased muscle BDNF expression and satellite cell proliferation (76). These data suggest that BDNF plays an important role in mediating satellite cell activation to muscle injury (11). However, BDNF overexpression in mouse muscle does not alter muscle mass (77). In line with this, BDNF is associated with muscle strength rather than muscle mass in humans (78).

Circulating BDNF levels are decreased in people with T2D independently of obesity and associated with impaired glucose homeostasis (8, 79). Reduced BDNF levels could contribute to beta cell dysfunction, reduced muscle oxidative capacity, and altered eating behavior promoting obesity and T2D. People with obesity carrying some specific variants of the BDNF gene achieve greater weight loss after bariatric surgery (80). We are not aware of any human BDNF analogs or agonists of its receptors. In contrast, circulating BDNF levels increase after exercise training in people with obesity and T2D and correlate positively with muscle strength in lower limbs (78). In older people, those with frailty (i.e., muscle weakness, slow walking speed, and low physical activity) have lower circulating BDNF levels than those without, and lower BDNF levels are associated with frailty regardless of age (81). Circulating BDNF levels correlate with exercise capacity and muscle strength, but not with muscle mass in people with heart failure (82). Thus, BDNF may be used as a biomarker of muscle strength rather than muscle mass and decreased BDNF levels in people with obesity and T2D may promote or reflect sarcopenic obesity.

Several studies, including ours, have shown decreased circulating BDNF levels three months to one year after bariatric surgery (54, 83, 84). In our study, decreased BDNF levels were associated with decreased steady-state estimate of beta cell function (i.e., HOMA2-%B), reflecting decreased compensatory hyperinsulinemia as expected from improved insulin sensitivity after bariatric surgery (54, 58). In contrast, we showed an upregulation of BDNF mRNA expression in vastus lateralis three months after bariatric surgery with an amplitude comparable to that observed after acute exercise (69). Interestingly, upregulation of muscle BDNF was associated with decreased insulin resistance (i.e., HOMA2-IR), suggesting a potential insulin-sensitizing effect of BDNF (54). Muscle BDNF is thought to act in an autocrine/paracrine manner to positively regulate muscle insulin sensitivity (68). Finally, upregulation of BDNF mRNA expression was negatively associated with the magnitude of fat-free mass loss, suggesting that upregulation of BDNF mRNA expression may be a mechanism aimed at maintaining muscle mass after bariatric surgery (54), whereas decreased circulating BDNF levels after bariatric surgery could reflect decreased muscle strength. Supervised exercise is recommended after bariatric surgery (85), as it has an additive effect to surgery in improving insulin sensitivity (32) and may improve muscle strength (86), cognition (87) and brain networks involved in regulating food intake (88). Higher circulating BDNF levels have been reported two years after bariatric surgery, together with sustained cognitive improvement, suggesting that changes in BDNF may be associated with cognitive improvement after bariatric surgery (87). The interaction between bariatric surgery and exercise may be highly relevant to BDNF actions as both upregulate BDNF in muscle. However, we are not aware of any studies that have examined the effects of both interventions combined on BDNF. Based on previous experimental evidence, we hypothesize that the reduction in circulating BDNF levels early after bariatric surgery may serve as a counter-regulatory response to sustained caloric restriction, weight loss and improved insulin sensitivity, whereas increased BDNF later after bariatric surgery may help improve obesity-related neurocognitive disorders and regulate food intake. However, this remains to be confirmed and further studies are needed to test these hypotheses.

### 3.3 Myostatin

MSTN, also known as growth differentiation factor 8 (GDF-8), is a member of the transforming growth factor (TGF)-beta superfamily that is secreted primarily by skeletal muscle and to a lesser extent by adipose tissue (4, 89). MSTN is expressed as a full-length precursor form, processed to latent MSTN by removal of its signal peptide, and subsequently activated by proteolytic cleavage by members of the bone morphogenetic protein (BMP)-1 family of metalloproteases (89). Mature MSTN is regulated extracellularly by several binding proteins, including FST (89). MSTN acts as a direct negative regulator of muscle mass through activin type II receptors and SMAD2-3 transcription factors, preventing pathological muscle hypertrophy (11, 89). Indeed, MSTN gene knockout in mice results in muscle hypertrophy, whereas MSTN overexpression in skeletal muscle results in muscle atrophy (89). On the one hand, MSTN activates FOXO-dependent transcription of atrogens (e.g., MURF-1, MAFbx/atrogin-1), leading to activation of muscle protein degradation and inhibition of myogenesis (11, 41). On the other hand, MSTN inhibits muscle protein synthesis by suppressing Akt-mediated mTORC1 activation (11, 90, 91). It has been suggested that MSTN may contribute to muscle wasting in diverse conditions in humans, including sarcopenic obesity (18, 92–97). In contrast, exercise decreases circulating levels and muscle expression of MSTN in humans, promoting increases in muscle mass and strength (13, 14, 98, 99).

Although MSTN is best known as a negative regulator of muscle mass, animal and cell models suggest that MSTN plays a pathogenic role in insulin resistance in obesity and T2D. Indeed, MSTN inhibition protects rodents from obesity and insulin resistance (100). Specifically, MSTN inhibition increases insulin-dependent and -independent glucose uptake and glycogen synthesis in muscle of normal chow-fed mice (100). This effect is likely mediated by increased GLUT1 and GLUT4 protein levels and increased muscle mass (100). MSTN inhibition also improves muscle glucose uptake in obese high-fat diet-fed mice by stimulating fatty acid metabolism and mitochondrial function, independently of muscle hypertrophy (101). In contrast, MSTN impairs insulin signaling, decreases AMPK activity, and inhibits GLUT4 expression in muscle cells, thereby impairing muscle glucose uptake and insulin sensitivity (102).

Circulating MSTN levels and muscle MSTN expression are typically higher in people with obesity and T2D than in healthy people (41, 103–106). Increased levels and phosphorylation of SMAD2-3 transcription factors have also been reported in muscle of people with obesity and T2D, altering myogenesis (20, 41, 103–105). There is a positive correlation between circulating MSTN levels or its muscle expression and insulin resistance in people with obesity and T2D (41, 104–106). Myotubes from insulin-resistant women with extreme obesity show increased MSTN secretion, suggesting that muscle is a source of increased circulating MSTN levels during obesity and T2D (41). In contrast, bimagrumab, a fully human monoclonal antibody that inhibits activin type II receptors, results in significant fat mass loss, increased lean mass, and improved insulin sensitivity in people with obesity and T2D (107, 108). Other compounds including Trevogrumab and Garetosmab are in the pipeline for muscle wasting conditions in humans (109).

Our research and that of others has shown that MSTN decreases at circulating and muscle levels after bariatric surgery (53, 54, 110). Kumar et al. have shown a 22% reduction in circulating MSTN levels six months after bariatric surgery in adolescents (53). Milan et al. have reported a 60% reduction in quadriceps MSTN mRNA expression eighteen months after bariatric surgery (110). We have also reported a significant reduction of MSTN in plasma (-32%) and vastus lateralis (-45% mRNA expression) three months after bariatric surgery, suggesting that decreased MSTN expression is responsible for decreased circulating MSTN levels (54). Like others, we did not find an association between changes in circulating or muscle MSTN and changes in glucose homeostasis as assessed by the HOMA test (53, 54), whereas decreased MSTN would be expected to improve muscle insulin sensitivity, independently of changes in muscle mass. In contrast, Milan et al. have found a positive correlation between decreased MSTN mRNA in vastus lateralis and decreased fat-free mass after bariatric surgery (110). We have also shown that decreased circulating MSTN levels were positively associated with decreased muscle mass after bariatric surgery, but inversely associated with sarcopenic obesity after bariatric surgery (55). In fact, a positive correlation between circulating MSTN levels and muscle mass or strength has been shown in various conditions, using recent immunoassays detecting active MSTN only (55, 111–118). Overall, these data suggest that decreased circulating MSTN levels after bariatric surgery may reflect decreased muscle and that circulating MSTN levels may be used as a biomarker of muscle mass. However, we also hypothesize that circulating MSTN does not play a major pathogenic role in muscle wasting after bariatric surgery. Decreased MSTN muscle expression and circulating levels after bariatric surgery may represent a counter-regulatory mechanism to preserve muscle mass during profound calorie and protein restriction (55, 110). Future studies should evaluate the relationship between MSTN and glucose homeostasis using more sophisticated methods to assess changes in muscle insulin sensitivity after bariatric surgery (30, 58).

### 3.4 Irisin

Irisin is produced by cleavage of the membrane protein fibronectin type III domain-containing protein 5 (FNDC5) (119). Although irisin is difficult to detect using commercially available ELISA kits, irisin has been detected in human plasma using mass spectrometry (120). Circulating irisin levels increase in response to acute exercise (10, 15, 121). However, FNDC5 mRNA is not induced after acute exercise in healthy human muscle (14). FNDC5 mRNA expression is higher in human muscle than in liver and adipose tissue, suggesting that muscle may be an important source of circulating irisin (122).

Irisin may have beneficial effects on skeletal muscle and beta cells. Mice fed a high-fat diet are protected against obesity and diabetes by irisin treatment (2, 119). Similarly, irisin treatment reduces body weight and blood glucose levels in rats with T2D (123). Muscle cells incubated with irisin show increased AMPK-mediated glucose uptake and fatty acid oxidation, suggesting a direct effect of irisin on muscle (10, 124, 125). In addition, irisin treatment improves GSIS in mice by increasing insulin biosynthesis and beta cell mass (126). Human beta cells and islets incubated with irisin are protected from palmitate-induced apoptosis, suggesting that irisin may protect beta cells from lipotoxicity in obesity (2, 126). In addition, irisin treatment induces a white-to-brown shift in mouse adipose tissue by increasing uncoupling protein (UCP) 1 expression, which increases whole-body energy expenditure (8, 119). Finally, muscle cells incubated with irisin show increased mitochondrial uncoupling and increased expression of UCP3, peroxisome proliferator-activated receptor gamma coactivator 1-alpha (PGC1A), and mitochondrial fusion genes, resulting in increased oxidative metabolism (127).

Several lines of evidence suggest that irisin promotes muscle hypertrophy (11). Irisin expression and secretion increase during myogenic differentiation in human primary myotubes (128). Irisin also induces myogenic differentiation of muscle cells by stimulating the expression of pro-myogenic genes (129). In addition, treatment of human primary myotubes with irisin results in muscle cell hypertrophy through downregulation of MSTN and upregulation of insulin-like growth factor I (IGF-I), a well-known anabolic factor (128). Mice injected with irisin exhibit muscle hypertrophy and improved grip strength due to activation of satellite cells and increased muscle protein synthesis (129). Finally, intraperitoneal irisin administration to aged mice mitigates age-related muscle atrophy (130). Overall, current experimental data on irisin suggests its potential for therapeutic purposes in sarcopenia. In humans, circulating irisin levels are positively correlated with muscle mass adjusted for body weight in postmenopausal women, and inversely associated with sarcopenia (131). In addition, irisin expression decreases with age in human muscle (130). These data suggest that irisin may positively regulate muscle mass in humans. Indeed, low circulating irisin levels are a predictive factor for sarcopenic obesity (132).

Some studies and a recent meta-analysis have reported lower circulating irisin levels in people with obesity and T2D than in healthy people (133–138). In these studies, circulating irisin levels were inversely correlated with body mass index (BMI) and insulin resistance in healthy and obese people (133–136). In contrast, several studies have reported higher circulating irisin levels in people with obesity (139, 140) and increased irisin secretion by insulin-resistant human myotubes (126). Consistent with these latter observations, FNDC5 mRNA expression in muscle is positively correlated with BMI (122). In addition, human visceral and subcutaneous adipose tissues secrete irisin and may contribute to higher circulating irisin levels in people with obesity (139, 140). Thus, decreased irisin could be associated with decreased oxidative metabolism and insulin sensitivity in muscle during obesity, whereas increased irisin could be interpreted as a counterregulatory mechanism to overcome insulin resistance. Overall, current experimental data on irisin suggests its potential for therapeutic purposes, especially in T2D and obesity, as irisin shares some metabolic effects with incretin hormones (141). However, considerable uncertainty remains regarding the reliability and accuracy of methods used to quantify circulating irisin, which may partly explain the discrepancies between studies (142) and possibly the cautious approach of pharmaceutical companies towards this myokine. We are not aware of any development of irisin analogs or agonists of its receptor.

Changes in circulating irisin levels after bariatric surgery show no discernible pattern (143), which may be related to discrepancies between commercial ELISA kits. Kumar et al. have reported increased circulating irisin levels in adolescents six months after bariatric surgery (53). However, no association was found with changes in HOMA-IR. In other studies, circulating irisin levels remained unchanged or even decreased from one month to one year after bariatric surgery in people with or without T2D at baseline (122, 136, 144, 145). In contrast, we and others have consistently found decreased FNDC5 mRNA expression in vastus lateralis 3-6 months after bariatric surgery (54, 122). Decreased irisin expression may be related to a transient decrease in mitochondrial function and oxidative metabolism in muscle early after bariatric surgery (31). Based on the documented anabolic effect of irisin on muscle cells, decreased irisin expression in muscle after bariatric surgery may contribute to muscle mass loss. However, the inconsistent results of circulating irisin levels after bariatric surgery preclude meaningful speculation about its role in this context in humans. Future studies should use validated ELISA techniques for irisin quantification to provide more reliable data.

### 3.5 Interleukin-6

Skeletal muscle is considered the main source of increased circulating IL-6 levels during exercise (1, 15). Contraction indeed upregulates IL-6 expression and secretion by human muscle cells and muscle tissue (1, 12–14, 50). Type II muscle fibers produce more IL-6 than type I muscle fibers during contraction (50).

IL-6 release from contracting muscle has been proposed to mediate some of the benefits of exercise on glucose homeostasis (2, 15). Indeed, acute infusion of IL-6 enhances insulin-stimulated glucose uptake in healthy humans, while chronic exposure to IL-6 increases glycogen synthesis and lipid oxidation through AMPK activation in primary human myotubes (146). In addition, acute IL-6 infusion stimulates lipolysis and fatty acid oxidation in muscle from healthy humans (147–149). IL-6 also enhances insulin secretion by increasing glucagon-like peptide-1 secretion from intestinal L cells and pancreatic alpha cells, suggesting that IL-6 may mediate muscle-gut-pancreas crosstalk (150).

The role of IL-6 in the regulation of muscle mass appears to be complex. IL-6 is produced transiently by growing muscle fibers and satellite cells (151). IL-6 gene deletion in mice blunts muscle hypertrophy in response to overload (151). Recovery of muscle mass after hindlimb suspension is also absent in IL-6 knockout mice because of reduced IGF-I expression and Akt/mTOR signaling in muscle (152). IL-6 appears to play a positive role in muscle growth, which would be consistent with elevated circulating IL-6 levels during exercise. In fact, genetic polymorphisms in the promoter region of the IL-6 gene that increase IL-6 levels have been linked to increased fat-free mass in men (153). The IL-6–STAT3 signalling may contribute to some muscle adaptations occurring after training, such as the induction of mitochondrial biogenesis and increased mitochondrial activity. However, this effect appears to be restricted to specific muscle tissues (153). In contrast, recombinant human IL-6 injected subcutaneously into rats causes muscle wasting (154). In addition, IL-6 overexpression from birth in mice reduces muscle growth during postnatal life, causing muscle atrophy (155). Similarly, a 3-hour infusion of recombinant human IL-6 in healthy people causes a significant reduction in muscle protein turnover and plasma amino acids (156). These observations suggest that locally produced IL-6 may have an anabolic effect on muscle in response to physiological stimuli (e.g., exercise), whereas chronically elevated levels of IL-6 may be detrimental to muscle mass. In fact, the effect of IL-6 on muscle mass and function may differ under physiological and pathological conditions that affect IL-6 signaling. Briefly, classical IL-6 signaling involves the binding of IL-6 to the membrane-bound IL-6 receptor alpha-subunit and glycoprotein 130 signal-transducing subunit. In contrast, IL-6 trans-signaling, which has emerged as the predominant pathway by which IL-6 promotes disease pathogenesis, involves the binding of complexes of IL-6 and the soluble form of IL-6 receptor to membrane-bound gp130 (157, 158).

Circulating IL-6 levels are chronically elevated in people with obesity and T2D but are still much lower than that in people with cancer and muscular dystrophy (8, 31, 155, 159, 160). Adipose tissue, rather than muscle, is considered the primary source of chronically elevated circulating IL-6 and other pro-inflammatory factors (e.g., TNF-a) levels in obesity (23, 24). However, human myotubes exposed to TNF-a show increased IL-6 secretion, suggesting that muscle may also contribute to increased circulating IL-6 levels in people with obesity and T2D (43). Mice fed a high-fat diet exhibit obesity-related systemic insulin resistance and elevated circulating IL-6 levels, while blockade of excessive IL-6 signaling improves systemic insulin sensitivity illustrated by upregulated skeletal muscle glucose uptake (161). In humans, subcutaneous administration of recombinant IL-6 induces dose-dependent increase in fasting blood glucose in healthy people, probably by stimulating glucagon release and/or by inducing peripheral insulin resistance (162), whereas IL-6 blockade improves glycated hemoglobin A1c (HbA1c) in people with T2D (163). In addition, skeletal muscle response to IL-6 is blunted in people with T2D. Indeed, acute IL-6 infusion/treatment does not increase glucose uptake in people with T2D or myotubes from people with T2D, conversely to healthy people (164, 165). Thus, on one hand, chronically elevated circulating IL-6 levels may contribute to impaired glucose homeostasis in obesity. On the other hand, acute IL-6 may be effective in preventing T2D by increasing muscle glucose uptake, an effect that is lost in people with T2D (10). In contrast, acute IL-6 infusion still stimulates lipolysis and fatty acid oxidation in muscle from people with T2D, as observed in healthy people (149). Interestingly, plasma levels and muscle expression of IL-6 increase robustly but transiently in response to acute exercise in people with T2D similarly to healthy people (166), suggesting that IL-6 may still exert beneficial actions in muscle during T2D. On the other hand, exercise training (including aerobic exercise and resistance training) decreases basal plasma IL-6 levels in people with T2D, which is associated with metabolic improvement (167, 168). Regarding muscle mass, human studies have suggested a possible association between elevated circulating IL-6 levels and age-related decline in muscle mass and strength (155). Indeed, older people with sarcopenia have higher IL-6 levels than those without, and IL-6 levels are associated with sarcopenia (159). In addition, higher circulating IL-6 levels in older people are associated with impaired muscle response to exercise training, and impaired physical performance (169). Therefore, chronically elevated IL-6 may be an important contributor to the decline in muscle mass, strength, and function in people with obesity and T2D (169), potentially contributing to sarcopenic obesity.

Kumar et al. have reported reduced circulating IL-6 levels in adolescents six months after bariatric surgery (53). However, no association was found with HOMA-IR. Similarly, Villarreal-Calderon et al. found reduced circulating IL-6 levels six months after bariatric surgery, while metabolic improvement was evident as early as three months (170). In contrast, Sajoux et al. reported unchanged circulating IL-6 levels after bariatric surgery (145). Overall, decreased circulating IL-6 may indicate decreased adipose tissue and muscle inflammation (171). However, we have reported increased IL-6 mRNA expression in vastus lateralis three months after bariatric surgery, although with a lower amplitude than after acute exercise (54). Although we did not find an association between changes in IL-6 mRNA expression and changes in glucose homeostasis or fat-free mass (54), we hypothesize that increased IL-6 mRNA expression in muscle after bariatric surgery may locally exert beneficial effects on glucose and lipid metabolism as well as anabolism.

### 3.6 Fibroblast growth factor 21

Fibroblast growth factor (FGF) 21, a member of the FGF19 subfamily, is mainly an hepatokine, but also an adipomyokine (56, 172). FGF21 binds to the beta-Klotho complex, a FGF co-receptor that is highly expressed in metabolically active tissues such as the pancreas (173). Several observations support the role of FGF21 in glucose homeostasis. FGF21 treatment in both regular chow- and high-fat diet-fed mice improves hepatic and muscle insulin sensitivity, by decreasing hepatocellular and intramyocellular diacylglycerol content (174). Systemic FGF21 administration lowers blood glucose levels in monkeys with diabetes (173). In addition, incubation of human myotubes with FGF-21 increases basal and insulin-stimulated glucose uptake by upregulating GLUT1 expression, suggesting a direct effect of FGF21 on muscle (175). FGF21 also protects beta cells from apoptosis, possibly by reducing glucolipotoxicity (173).

FGF21 expression is very low in healthy muscle (176). However, cellular stressors including fasting, endoplasmic reticulum stress, mitochondrial myopathies, and metabolic disorders induce FGF21 release from muscle (176, 177). Muscle-specific FGF21 knockout mice have similar muscle fiber size than control mice, indicating that FGF21 does not control muscle mass under basal conditions (176). In contrast, FGF21 knockout mice are protected against starvation-induced muscle atrophy, whereas FGF21 overexpression in muscle induces muscle atrophy by activating autophagy (176). Similarly, systemic FGF21 treatment in female mice induces muscle atrophy, possibly through glucocorticoid signaling (178). In contrast, skeletal muscle-specific Akt1 transgenic mice which are characterized by muscle fiber hypertrophy exhibit increased FGF21 muscle expression and circulating levels (179). Taken together, these data suggest that muscle is a source of circulating FGF21 and that FGF21 promotes muscle atrophy in the context of cellular stress.

Increased circulating FGF21 levels have been reported in people with prediabetes and T2D, which may be a consequence of metabolic imbalance and/or FGF21 resistance (173, 175, 180). Circulating FGF21 levels correlate with fasting insulin, insulin resistance, and BMI in people with T2D but not in healthy people (175). However, muscle FGF21 mRNA expression is similar in people with T2D and healthy people (175), suggesting that increased circulating FGF21 levels do not originate from muscle. Plasma FGF21 levels increase after acute exercise (15). However, FGF21 analogs have shown little or no efficacy in improving body weight, blood glucose, and HbA1c in people with obesity and T2D (181). Regarding muscle mass, older people with sarcopenia have higher circulating FGF21 levels than those without (182). In addition, FGF21 levels were found to be associated with an increased likelihood of sarcopenia, low muscle mass, and low muscle strength, independent of sex, age, and BMI (182). However, a meta-analysis found no difference in circulating FGF21 levels between people with and without sarcopenia, and no strong correlation between the onset of sarcopenia and circulating FGF21 levels (183). Nevertheless, these data suggest that elevated FGF21 levels in people with obesity may be associated with sarcopenic obesity.

A steep increase in circulating FGF21 levels has been observed within weeks after bariatric surgery, followed by a slight decrease over time (184–186). These changes may be related to initial muscle wasting in response to caloric restriction, which tends to decrease over time. No association was found between changes in circulating FGF21 levels and HOMA-IR early after bariatric surgery (184). At longer term, people with T2D who had undergone bariatric surgery 12 years earlier exhibited similar circulating FGF21 levels than those who had not undergone bariatric surgery, despite lower BMI (187). Increased FGF21 expression in muscle after bariatric surgery would be expected to improve muscle insulin sensitivity, possibly through intramyocellular lipid depletion and restored adiponectin expression in adjacent adipocytes (174, 188). However, muscle data in humans are currently lacking to establish that changes in circulating FGF21 after bariatric surgery originate from muscle.

### 3.7 Meteorin-like protein

Meteorin-like protein (Metrnl) (also known as subfatin) is a neurotrophic factor homologous to meteorin that is abundant in cerebrospinal fluid and plays a role in neuroblast migration and neuroprotection (189). In addition, Metrnl is a cold- and caloric restriction-induced adipokine and a contraction-induced myokine that has been detected in conditioned media from muscle cells (45, 189–191). Resting muscle cells express only low levels of Metrnl, while its expression is upregulated by exercise along with PGC1A expression in human muscle tissue (13, 191). Consistently, plasma Metrnl levels increase after acute and chronic exercise (15).

Several observations support that Metrnl may have beneficial effects on glucose homeostasis. Metrnl treatment in mice increases the expression of genes associated with beige fat thermogenesis, as well as the expression of anti-inflammatory cytokines in white adipose tissue (191). In addition, Metrnl promotes adipocyte differentiation and lipid storage, likely by acting through induction of the transcription factor peroxisome proliferator-activated receptors (PPAR) gamma, the key regulator of adipocyte differentiation (191). Adipocyte-specific Metrnl knockout in mice exacerbates high-fat diet-induced insulin resistance, whereas adipocyte-specific transgenic Metrnl overexpression prevents high-fat diet-induced insulin resistance (192).

Data on circulating Metrnl levels in people with obesity and T2D are conflicting (189). Some studies have reported higher circulating Metrnl levels in people with obesity and T2D (193), while other studies have found lower Metrnl levels which negatively correlated with HbA1c (189, 194).

Data on changes in circulating Metrnl levels after bariatric surgery are also conflicting. Pellitero et al. found increased circulating Metrnl levels 12 months after bariatric surgery, which were inversely associated with HOMA-IR (194). These data suggest a potential insulin-sensitizing effect of Metrnl, which is consistent with experimental observations (192). In contrast, Schmid et al. reported decreased circulating Metrnl levels within days of bariatric surgery, followed by a progressive return to baseline (189). Decreased circulating Metrnl levels after bariatric surgery were also observed in rats, while Metrnl expression was increased in muscle and white adipose tissue (195). In contrast, liver Metrnl expression is reduced in humans after bariatric surgery, suggesting that tissues other than muscle and adipose tissue may contribute to changes in circulating Metrnl levels after bariatric surgery (196). Muscle data in humans are currently lacking to establish that changes in circulating Metrnl after bariatric surgery originate from muscle.

### 3.8 Myonectin

Myonectin (also known as erythroferrone or CTRP15) is a C1q/TNF-related protein that is predominantly expressed by skeletal muscle and is robustly secreted by muscle cells (197). Higher myonectin mRNA expression was found in slow-twitch and oxidative muscles compared to fast-twitch and glycolytic muscles in mice (197). Myonectin is tightly regulated by metabolic state, with fasting suppressing circulating myonectin and refeeding dramatically increasing both circulating and muscle mRNA levels (197). Similarly, diet-induced obesity decreases circulating and muscle mRNA levels of myonectin, whereas voluntary exercise increases both circulating and muscle mRNA levels (197). Administration of myonectin to mice reduces circulating levels of free fatty acids without altering adipose tissue lipolysis and enhances fatty acid uptake in cultured adipocytes and hepatocytes (197). Consistent with this, myonectin knockout mice have impaired lipid clearance from the blood and insulin resistance when fed a high-fat diet (198). In contrast, myonectin does not lower blood glucose levels (197). Collectively, these data suggest that myonectin links skeletal muscle to lipid homeostasis in liver and adipose tissue in response to changes in energy status (197).

In addition, myonectin may mitigate muscle atrophy (11). Indeed, disruption of myonectin exacerbates muscle atrophy and mitochondrial dysfunction related to age, sciatic denervation and dexamethasone in mice (199). In contrast, treatment with myonectin attenuates or suppresses muscle atrophy in these murine models via activation of AMPK/PGC1A signaling (199). Slow-twitch muscle fibers exhibit higher myonectin expression than fast-twitch muscle fibers, suggesting that myonectin may control mitochondrial biogenesis and muscle function (197).

Data on circulating myonectin levels in people with obesity and T2D are conflicting. Several studies have found lower circulating myonectin levels in people with obesity compared to healthy people, as well as a negative association between myonectin and indicators of metabolic risk, including BMI, visceral fat content, and indexes of insulin resistance (200, 201). In contrast, other studies have found higher circulating myonectin levels in people with obesity and T2D, as well as a positive association between myonectin, fat mass, and insulin resistance (202). Exercise training increases circulating myonectin levels in people with obesity, as observed in mice (15, 203).

Data on myonectin after bariatric surgery is limited. Li et al. demonstrated increased circulating myonectin levels six months after bariatric surgery (200). Although circulating myonectin levels were negatively associated with HOMA-IR at baseline, no association with HOMA-IR after bariatric surgery was reported in this study. In contrast, Butler et al. showed that people who had undergone bariatric surgery had similar circulating myonectin levels to those who had not undergone bariatric surgery, even though BMI was lower after bariatric surgery (187). However, these data were obtained 12 years after bariatric surgery. Increased myonectin levels after bariatric surgery could be interpreted as a mechanism to promote lipid uptake in tissues such as liver and muscle during caloric restriction. In addition, increased circulating myonectin levels after bariatric surgery may contribute to attenuating the decline in muscle mass and mitochondrial function observed at early time points after bariatric surgery. However, to our knowledge, associations between changes in myonectin and changes in muscle mass after bariatric surgery have never been reported. In our opinion, myonectin is an interesting candidate for future studies because its changes at circulating and muscle levels may be related to changes in lipid metabolism and muscle mass after bariatric surgery.

### 3.9 Apelin

Apelin (APLN) is a 36 amino acid peptide and the endogenous ligand of the G-protein coupled receptor APJ receptor. APLN is secreted by human muscle cells and various tissues, including adipose tissue (204, 205). APLN expression increases in response to insulin in adipocytes and to contraction in human muscle cells and tissue (204, 206). Muscle APLN expression increases after exercise and is associated with improved whole-body insulin sensitivity (204). In addition, treatment of APLN-null or obese insulin-resistant mice with APLN restores glucose tolerance by improving muscle glucose uptake and oxidation (207–209).

In addition, APLN plays a critical role in muscle physiology during aging (210). In the heart, APLN deficiency leads to premature cardiac aging (210, 211). In skeletal muscle, APLN deficiency (whole body and muscle-specific) or APLN receptor deficiency leads to muscle atrophy and functional changes in mice (210). In contrast, APLN treatment in aged mice reverses age-related sarcopenia by improving various processes associated with muscle rejuvenation in an AMPK-dependent manner, leading to muscle hypertrophy and strength recovery (210). In young and aged primary human myotubes, APLN treatment induces the activation of anabolic pathways that promote protein synthesis (AKT, mTOR, P70S6K and 4E-BP1). In addition, APLN inhibits the activation of age-related proteolysis players such as FOXO3a in aged human primary myotubes (210). Furthermore, APLN treatment induces muscle cell differentiation, thereby enhancing myogenesis (210). These data suggest that the development of agonists for the APLN receptor warrants further investigation as pharmacological strategies for sarcopenia.

Circulating APLN levels are higher in people with obesity and T2D and inversely associated with insulin sensitivity, suggesting either APLN resistance or increased secretion from adipose tissue (205, 206, 212, 213). Indeed, muscle APLN expression is similar between healthy people and those with T2D (204). In contrast, adipose tissue APLN expression is increased in people with T2D, suggesting that adipose tissue is responsible for increased circulating APLN levels in obesity and T2D (205, 208). Plasma APLN levels are independently associated with age-related sarcopenia (210). Furthermore, muscle APLN production in response to exercise is reduced with aging (210). Indeed, aging is associated with loss of skeletal muscle APLN and APLN receptor mRNA expression, as observed in mice and cultured primary human myotubes. Thus, APLN could be used as a biomarker of sarcopenia (210).

Decreased circulating APLN levels have been reported seven to twelve months after bariatric surgery and decreased APLN levels are associated with improved insulin sensitivity (205, 212, 213). Decreased APLN levels may also reflect or contribute to skeletal muscle mass loss after bariatric surgery, but data are lacking to confirm this hypothesis. We have shown that APLN mRNA expression in vastus lateralis is unchanged three months after bariatric surgery, which does not exclude changes in other muscle groups (54). In contrast, APLN expression in adipose tissue is reduced after bariatric surgery, which likely contributes to reduced circulating APLN levels and may be associated with improved glucose homeostasis (205). Thus, current data suggest that decreased circulating APLN contributes to or reflects improved insulin sensitivity after bariatric surgery. However, it has not been established that the changes observed at the circulating level originate from muscle.

### 3.10 Secreted protein acidic and rich in cysteine

Secreted protein acidic and rich in cysteine (SPARC or osteonectin) is a glycoprotein that regulates cell-extracellular matrix (ECM) interactions and exerts profibrotic effects in various tissues, including muscle (214, 215). In addition, SPARC is expressed in satellite cells and stimulates myogenesis (216–218). As a result, SPARC knockout mice have decreased muscle mass and impaired force recovery (216, 217, 219). In fact, reduced SPARC expression in mouse muscle upregulates atrogens, increases TGF-beta signaling, and decreases myofiber size, suggesting that SPARC deficiency leads to muscle atrophy (218). In contrast, SPARC treatment in young rats was found to be effective in promoting myogenesis (218). These data suggest that SPARC plays a role in the regulation of satellite cell function and protects against muscle atrophy. Paradoxically, SPARC is expressed in myotubes, myofibers, and satellite cells in several inherited and idiopathic muscle wasting diseases (216). In fact, transient increase in SPARC expression occurs during muscle regeneration and correlates with the expression of myogenic muscle factors (220). Therefore, increased SPARC expression in myopathies can be interpreted as a mechanism aimed at muscle repair.

Circulating levels and muscle mRNA expression of SPARC are higher in people with obesity and T2D than in lean people (214). Similarly, circulating levels of MMP-2 (72 kDa type IV collagenase), a key metalloproteinase involved in the removal of excess ECM (215), are higher in people with obesity than in lean people (145). People with sarcopenia have higher circulating SPARC levels than those without (221). This also suggests that SPARC can serve as a potential biomarker for sarcopenia.

Lee et al. have shown decreased circulating SPARC and MMP-2 levels nine months after bariatric surgery (215). Changes in circulating SPARC levels, but not MMP-2, correlated significantly with changes in HOMA-IR, suggesting that reduced SPARC is associated with improved insulin sensitivity after bariatric surgery (215). In contrast, Sajoux et al. have reported increased circulating MMP-2 levels six months after bariatric surgery (145), which may contribute to a healthier ECM phenotype in muscle. Notably, resistance exercise also increases circulating MMP-2 levels in humans (145). These data suggest that SPARC and MMP-2 may play opposing roles in the ECM remodeling during obesity and after bariatric surgery, thereby regulating muscle insulin sensitivity. Despite the documented effects of SPARC on muscle mass, data are lacking to determine its potential contribution to changes in muscle mass after bariatric surgery.

### 3.11 Follistatin

FST is a glycoprotein mainly secreted by the liver that is also detected in conditioned media from human myotubes, which qualitfies FST as a myokine (40). FST inhibits several members of the TGF-beta family, including MSTN (222). Muscles of FST heterozygous knockout mice exhibit reduced size and force production, and impaired muscle repair (222). In contrast, FST overexpression induces muscle hypertrophy through proliferation of satellite cells (i.e., muscle fiber precursors) and inhibition of MSTN and activin A (222). Circulating FST levels are usually higher in people with insulin resistance and T2D than in healthy people (223, 224). FST and MSTN secretion are both increased by primary myotubes from people with obesity and T2D, suggesting that muscle may contribute to increased circulating FST levels (40). In addition, increased FST at circulating and muscle levels could be interpreted as a compensatory mechanism to counteract increased MSTN during obesity. However, circulating FST levels are negatively associated with muscle mass, strength, and function in older women, suggesting that FST may be used as a biomarker of sarcopenia (225, 226).

The effects of bariatric surgery on circulating FST levels are inconsistent. Some studies have reported increased circulating FST levels after bariatric surgery (227, 228), while others have reported decreased or unchanged levels (228–230). Changes in circulating FST after bariatric surgery are likely of hepatic origin, as the liver is the major contributor to FST levels both at rest and during exercise (231). Nevertheless, Dantas et al. have shown that exercise training combined with bariatric surgery further decreases TGF-beta signaling, MSTN expression, SMAD 2-3 phosphorylation but increases FST expression in muscle, compared to bariatric surgery alone, which decreases FST expression (32). Thus, the interaction between bariatric surgery and exercise may be relevant to the auto- and paracrine actions of FST. Increased FST expression in muscle after bariatric surgery may help preserve muscle mass or muscle strength (85), although this remains to be confirmed.

### 3.12 Other myokines

Like SPARC and MMP-2, other myokines are involved in ECM remodeling, a process that is altered in muscle of people with obesity and T2D (232) but improved by bariatric surgery (32). However, the role of these myokines in ECM remodeling of human muscle after bariatric surgery remains to be determined. ADAM metallopeptidase with thrombospondin type 1 motif 9 (ADAMTS9) is a secreted metalloprotease active against proteoglycans (e.g. versican, aggrecan) (233). Computational analysis of the human secretome predicts that ADAMTS9 is a myokine (3, 5). The ADAMTS9 rs4607103 C allele is one of several genetic variants proposed to increase the risk of T2D through impaired insulin sensitivity (233). This variant is associated with increased expression of secreted ADAMTS9, decreased insulin sensitivity and decreased expression of mitochondrial markers in human muscle (233). Consistent with this, mice selectively lacking Adamts9 in skeletal muscle have improved insulin sensitivity, whereas overexpression of Adamts9 in muscle leads to impaired mitochondrial function, accumulation of harmful lipid intermediates, and impaired insulin signaling (233). To our knowledge, changes in circulating ADAMTS9 levels after bariatric surgery have not been reported. We have reported that ADAMTS9 mRNA expression increases in vastus lateralis three months after bariatric surgery (54). However, we did not find an association with changes in glucose homeostasis as assessed by the HOMA test (54), and whether ADAMTS9 would play a positive or negative role in ECM remodeling in the context of bariatric surgery is currently unknown.

Tripartite motif containing 72 (TRIM72, also known as mitsugumin or MG53) is a striated muscle-specific E3 ligase that promotes ubiquitin-dependent degradation of the insulin receptor and IRS-1, inducing muscle insulin resistance (234, 235). MG53 is detected in conditioned media from human muscle cells (5). Using perfused mouse muscle, Wu et al. showed that high glucose and insulin levels, conditions mimicking obesity and T2D, induce MG53 release by muscle (234). Similarly, oral administration of glucose increased circulating MG53 and blood glucose levels in healthy humans (234). In addition, systemic administration of recombinant MG53 causes systemic insulin resistance and metabolic syndrome in mice, by blocking the insulin receptor (234). However, we reported increased MG53 mRNA expression in vastus lateralis three months after bariatric surgery, but we did not find an association with changes in glucose homeostasis as assessed by the HOMA test (54). A better understanding of the role of MG53 in regulating muscle insulin sensitivity after bariatric surgery may provide new therapeutic avenues. Of note, no reliable ELISA kits are available for the quantification of circulating human MG53 (234).

Angiogenin and osteoprotegerin are type II muscle specific myokines that are more highly expressed and secreted by human triceps than soleus myotubes (49). Both myokines exert beta cell protective effects alone or against proinflammatory cytokines, such as reduced beta cell apoptosis and preserved GSIS (49). These myokines may mediate muscle-pancreas crosstalk. Osteoprotegerin acts as a decoy receptor for TNFSF11/RANKL and TNFSF10/TRAIL (236), thereby neutralizing their inflammatory effects, whereas angiogenin is a secreted protein involved in protein synthesis and angiogenesis (237). We have reported increased angiogenin and osteoprotegerin mRNA expression in vastus lateralis three months after bariatric surgery, but we did not find any association with changes in glucose homeostasis as assessed by the HOMA test (54). These myokines may contribute to improved beta cell function after bariatric surgery through anti-inflammatory and possibly pro-angiogenic effects.

IL-8, another member of the interleukin family, is a myokine that is potentially involved in angiogenesis in muscle (145). Circulating IL-8 levels increases in response to exercise (15, 145). IL-8 is also increased in conditioned media from insulin-resistant human myotubes exposed to pro-inflammatory factors (e.g., TNF-a) (43). Circulating IL-8 levels are lower in people with obesity than in lean people and non-significantly increased early after bariatric surgery (2-3 months), with a tendency to drift back to baseline levels over the longer term (4-6 months) (145). Notably, IL-8 secretion by primary human myotubes increases after bariatric surgery, suggesting that muscle can contribute to increased circulating IL-8 levels (238).

We have also identified xylosyltransferase 1 (XYLT1), leucine-rich repeat-containing G protein-coupled receptor 5 (LGR5), and serine protease inhibitor Kazal-type 5 (SPINK5) as putative myokines that may contribute to improved glucose homeostasis after bariatric surgery (54). mRNA expression of these putative myokines was indeed increased in vastus lateralis three months after bariatric surgery and associated with improved insulin sensitivity (54). XYLT1 is expressed in many tissues, including pancreas, muscle, and adipose tissue, and encodes xylosyltransferase 1, which is involved in the biosynthesis of extracellular matrix glycosaminoglycan chains (61). LGR5 is highly expressed in skeletal muscle and encodes the leucine-rich repeat-containing G protein-coupled receptor 5 involved in the canonical Wnt signaling pathway, which is associated with myogenic differentiation (61, 239). SPINK5 encodes serine peptidase inhibitor Kazal type 5, which is a serine protease inhibitor involved in anti-inflammatory protection (61). These putative myokines may therefore be involved in ECM remodeling, improved myogenesis and decreased inflammation in muscle after bariatric surgery. However, few data are available on these proteins and glucose homeostasis. XYLT1 and LGR5 variants have been reported to be associated with T2D in genome-wide association studies, suggesting a link between these proteins and glucose homeostasis (240–242). To the best of our knowledge, these proteins have not been reported in conditioned media from muscle cells, so we cannot confirm that they are truly myokines. Mechanistic studies are needed to characterize their role in the regulation of glucose homeostasis.

## 4 Discussion

### 4.1 Changes in myokines associated with changes in glucose homeostasis after bariatric surgery

Improved glucose homeostasis after bariatric surgery is characterized by improved systemic and tissue-specific insulin sensitivity as well as recovery of GSIS (58). These processes contribute to the remission of T2D after bariatric surgery (30, 57, 58). Bariatric surgery is associated with changes in circulating levels and muscle expression of several myokines (**Table 1**). The myokines identified in this review are all potentially involved in the regulation of glucose homeostasis in humans, based on their known metabolic effects (**Figure 1**). However, for most of them, no association was found between changes in their circulating levels or muscle expression and changes in glucose homeostasis after bariatric surgery. Furthermore, for most of them, only changes at the circulating level have been reported. Nevertheless, changes in certain myokines, including upregulation of muscle BDNF mRNA and increased circulating Metrnl levels, are associated with improved insulin sensitivity after bariatric surgery (54, 194). In addition, decreased circulating SPARC and APLN levels are associated with improved insulin sensitivity (212, 213, 215). These data suggest that changes in these specific myokines may mediate the insulin-sensitizing effect of bariatric surgery. Emerging evidence also suggests that bariatric surgery-induced changes in certain myokines, such as decreased circulating levels of BDNF (54), may be associated with changes in beta cell function after bariatric surgery. BDNF may therefore mediate muscle-pancreas crosstalk after bariatric surgery. Finally, myonectin is an interesting candidate for future studies as this myokine may regulate lipid metabolism after bariatric surgery. In conclusion, despite limited data, some myokines appear to be potential mediators of changes in glucose homeostasis after bariatric surgery.

### 4.2 Changes in myokines associated with changes in muscle mass after bariatric surgery

Loss of skeletal muscle mass is one of the most notable but unintended changes occurring after bariatric surgery (33). Bariatric surgery is associated with changes in circulating levels and muscle expression of myokines that may be involved in the regulation of muscle mass and strength in humans (**Table 1**). The results of experimental research indicate that FST, irisin, IL-6, BDNF, myonectin, SPARC, and APLN promote muscle hypertrophy and/or stimulate myogenesis, whereas MSTN and FGF21 can induce muscle atrophy (11) (**Figure 1**). However, for most of these myokines, their role in the regulation of muscle mass and strength in humans remains speculative, and consistent data after bariatric surgery are lacking. Among the myokines identified in this review, MSTN is undoubtedly the myokine most specifically involved in regulating muscle mass and best documented following bariatric surgery. Counterintuitively, we and others have shown that circulating levels of MSTN and its muscle expression decrease after bariatric surgery and correlate positively with muscle mass in this context (53–55, 110). Decreased MSTN signaling after bariatric surgery could be a protective mechanism for muscle mass during profound calorie and protein restriction. As for glucose homeostasis, some myokines appear to be potential mediators of changes in muscle mass after bariatric surgery, although data are limited.

### 4.3 Challenges in the study of myokines after bariatric surgery

There are several limitations to studying the contribution of myokines to changes in glucose homeostasis and muscle mass after bariatric surgery. Skeletal muscle has the potential to be a primary source of peptides at the circulating level because skeletal muscle is the largest tissue in the human body (90). However, most peptides known as myokines are not specifically secreted by skeletal muscle. The relative contribution of skeletal muscle in the regulation of circulating levels of such peptides is difficult to determine, especially in humans. Indeed, there is a large overlap between adipokines, hepatokines, cytokines, and myokines (12, 56). Thus, changes in circulating levels of peptides known as myokines after bariatric surgery may not originate from muscle. Analysis of the arteriovenous difference would be required to establish that muscle is the main source of the changes in myokines observed in the circulation (243), but this has limited applicability. The discrepancy between changes in circulating levels and gene expression of certain myokines, as observed for BDNF in our study (54), suggests that some myokines may act in an autocrine/paracrine rather than in an endocrine manner. Consequently, changes in muscle mRNA levels do not necessarily translate into changes in circulating levels (12). However, obtaining muscle samples before and after bariatric surgery in humans to assess changes at the mRNA level remains challenging.

Some myokines are preferentially expressed and secreted by certain muscle fibers (49, 50). The type of muscle analyzed, and more specifically the proportion of type I and type II muscle fibers, may therefore influence the myokine response to bariatric surgery. Single-cell analysis (e.g. single-cell RNA-sequencing) would offer greater precision in analyzing the response of type I and type II myotubes to bariatric surgery, as shown for exercise (244).

The muscle bed includes myofibers and muscle satellite cells, as well as fibroadipogenic progenitor cells, myoendothelial and endothelial cells, neurons, and immune cells (245). These different cell types influence all measurements made on intact tissue (245). It is therefore difficult to determine the specific contribution of muscle fibers to changes in myokine gene and protein expression using muscle samples, although they capture the *in vivo* situation. Primary human muscle cell culture offers the advantage of isolating muscle cells that have been shown to retain the metabolic characteristics of the donor (42, 246). However, myokines are expressed at low levels at rest, making their detection difficult in conditioned media under basal conditions. In addition, mass spectrometry, the most accurate and specific method currently available for secretome analysis, requires serum starvation, a cellular stressor that can alter muscle cell viability, metabolism, and differentiation (247). On the other hand, cell culture models have disadvantages such as a lower degree of muscle cell differentiation, lack of nerve stimulation and microenvironment, and potential contamination by fibroblasts, which may limit the translation of *in vitro* findings to the *in vivo* situation (247). To our knowledge, only one study has investigated changes in myokine secretion from human primary myotubes after bariatric surgery (238).

Regarding the effects of bariatric surgery on myokines, the current findings require further validation in larger studies and in more diverse populations. Most studies investigating the effects of bariatric surgery include premenopausal and Caucasian women, whereas age, sex, ethnicity, and body composition influence the muscle transcriptome, proteome, and phenotype (248, 249). In addition, most people who undergo bariatric surgery remain in the obese range early after bariatric surgery (BMI > 30kg/m²), which may still result in “higher or lower than normal” myokine levels. The relative contribution of bariatric surgery itself versus weight loss and lifestyle changes (e.g., increased exercise) in inducing changes in myokines after bariatric surgery is difficult to determine. In addition, the observed discrepancies in myokine levels and response to bariatric surgery between studies may be due to differences in assay methods or different physical activity levels. Overall, the extent to which muscle-derived factors alter circulating levels to influence distant organs remains to be determined in the context of bariatric surgery (2).

The fact that some myokines may exert opposite effects in healthy conditions and in metabolic diseases (e.g., IL-6) is another difficulty in inferring the expected effects of changes in specific myokines on glucose homeostasis and muscle mass. In addition, the effects of certain myokines are observed in the context of cellular stress (e.g., inflammation, fasting, and exposure to palmitic acid) because their expression and secretion are otherwise low under basal conditions.

The method used to assess glucose homeostasis also influences the results of human studies. Muscle insulin sensitivity can be assessed *in vivo* using several validated techniques. The hyperinsulinemic-euglycemic clamp (HEC) is the gold standard method for assessing whole-body glucose uptake *in vivo* (i.e., M-value), which is primarily dependent on muscle insulin sensitivity (250). However, the HEC is a complex procedure with limited daily applicability. Therefore, HOMA-IR is often used as an acceptable surrogate marker of muscle insulin sensitivity, as HOMA-IR correlates reasonably well with M in various populations (251). HOMA-IR can be easily calculated from fasting plasma insulin and glucose concentrations using the following formula [fasting glucose (mmoL/L)*fasting insulin (mUI/L)/22.5] (252) or a more sophisticated mathematical model (i.e., HOMA-2) (253). The HOMA-2 calculator can also provide a steady state estimate of beta cell function (HOMA2-B%). In most of the studies discussed in this review, glucose homeostasis was assessed using the HOMA test. We hypothesize that studies with a better assessment of muscle insulin sensitivity using the gold standard HEC may yield different results.

Finally, the muscle secretome is not limited to peptides (2, 6, 15), and the potential contribution of muscle metabolites and microRNAs to changes in glucose homeostasis and muscle mass after bariatric surgery requires further studies.

## 5 Concluding remarks

It is now well established that bariatric surgery alters the circulating levels and muscle expression of several myokines. Changes in some of them, including BDNF, Metrnl, SPARC, and APLN are associated with improved glucose homeostasis, particularly improved insulin sensitivity, after bariatric surgery. However, whether skeletal muscle is the source of the reported changes in circulating levels of these peptides has not been established. Myonectin is an interesting candidate for future studies, as this myokine may regulate lipid metabolism and muscle mass after bariatric surgery. Finally, MSTN is associated with changes in muscle mass after bariatric surgery and may serve as a biomarker and as a counter-regulatory mechanism of muscle mass loss in this context. Identification of myokines that regulate glucose homeostasis and muscle mass after bariatric surgery would provide novel therapeutic targets and biomarkers for obesity, T2D, and sarcopenia.
